# A lightweight cross-scale feature fusion model based on YOLOv8 for defect detection in sewer pipeline

**DOI:** 10.1371/journal.pone.0330677

**Published:** 2025-08-21

**Authors:** Ruibo Sha, Zhifeng Zhang, Xiao Cui, Qingzheng Mu

**Affiliations:** Software Engineering College, Zhengzhou University of Light Industry, Zhengzhou, Henan, China; Tongji University, CHINA

## Abstract

Sewer pipeline defect detection is a critical task for ensuring the normal operation of urban infrastructure. However, the sewer environment often presents challenges such as multi-scale defects, complex backgrounds, lighting changes, and diverse defect morphologies. To address these issues, this paper proposes a lightweight cross-scale feature fusion model based on YOLOv8. First, the C2f module in the backbone network is replaced with the C2f-FAM module to enhance multi-scale feature extraction capabilities. Second, the HS-BiFPN module is adopted to replace the original structure, leveraging cross-layer semantic fusion and feature re-weighting mechanisms to improve the model’s ability to distinguish complex backgrounds and diverse defect morphologies. Finally, DySample is introduced to replace traditional sampling operations, enhancing the model’s ability to capture details in complex environments. This study uses the Sewer-ML dataset to train and evaluate the model, selecting 1,158 images containing six types of typical defects (CK, PL, SG, SL, TL, ZW), and expanding the dataset to 1,952 images through data augmentation. Experimental results show that compared to the YOLOv8n model, the improved model achieves a 3.8% increase in mAP, while reducing the number of parameters by 35%, floating-point operations by 21%, and model size by 33%. By improving detection accuracy while achieving model lightweighting, the model demonstrates potential for application in pipeline defect detection.

## Introduction

As an important part of urban infrastructure, sewer pipeline are mainly responsible for collecting and transporting domestic sewer, industrial wastewater, and rainwater, and their operation directly affects the level of urban development, public health, and environmental quality. Therefore, ensuring the efficient operation of the sewer pipeline system is crucial for the sustainable development of cities [1]. With continued economic growth and accelerated urbanization, China’s urban sewerage network system has made significant progress in construction and development [2]. However, in recent years, the management of sewer pipeline generally has the problem of “heavy construction, light care”, resulting in structural damage, corrosion, clogging, and other defects of the pipeline gradually appearing. If these hazards are not identified and addressed in a timely manner, they may lead to more serious environmental pollution and damage to facilities [3]. Therefore, it is particularly important to check the internal condition of sewer pipeline regularly to assess the type and location of defects promptly [4] and to take appropriate countermeasures such as pipeline maintenance, repair, or replacement of severely damaged parts [5].

Closed Circuit Television (CCTV) detection technique have become a widely used means of inspection and maintenance of sewer pipeline. Typically, robots deployed with camera equipment and lighting devices move inside the pipeline and record videos in real time, which are used to assess the structural condition of the pipeline. This technique is widely recognized for its ease of operation and low cost [6, 7]. Video allows technicians to observe the condition of defects within the pipeline, such as cracks, collapses, and deposits [8]. Although CCTV detection technique has been widely used, the traditional manual interpretation method is not only time-consuming and labor-intensive but also susceptible to operator experience and subjective judgment, resulting in biased identification results [9]. For example, cracks may be misjudged as fractures. Although technicians can refer to standards such as the Pipeline Assessment Certification Program (PACP) as an aid [10], this human-based inspection method makes it difficult to meet the demands of efficient and accurate modern inspections.

With the continuous development of computer vision technology and artificial intelligence technology, sewer pipeline detection methods are also evolving. According to the different methods of feature design, they can be categorized into: traditional image processing methods and deep learning-based methods. Some have applied traditional computer vision and image processing techniques to identify defects in videos and images of wastewater pipelines. L. Soibelman *et al*. proposed a SIFT-based defect detection method for wastewater pipelines, which realizes automatic detection of image defects through a three-step automated detection process of local scale, orientation, and illumination invariant features [11]. M.R. Halfawy *et al*. proposed a defect recognition method based on Histogram of Oriented Gradients (HOG) and Support Vector Machines (SVM) for automatic detection and classification of defects in municipal sewers, especially tree root intrusion problem. The algorithm extracts the region of interest (ROI) by image segmentation and classifies the defects using SVM classifiers to improve the time-consuming, costly, and error-prone traditional CCTV inspection process [12]. Later M.R. Halfawy *et al*. proposed a recognition method based on an optical flow algorithm, Haar features, and a multi-class support vector machine, aiming to improve the efficiency of video review, quality control, and historical data extraction [13]. S. Moradi *et al*. proposed a pipeline defects detection method using Hidden Markov Model. The method uses a Hidden Markov Model (HMM) to model proportional data to detect and locate anomalies in real-time, including modeling normal conditions and identifying outliers with this model [14]. Traditional vision-based feature extraction methods often require the design of complex feature extractors with poor robustness. In contrast, deep learning-based vision algorithms have demonstrated superior performance in most computer vision tasks (e.g., image classification, target detection, and segmentation) [15].

With the rapid development of deep learning technology, deep learning algorithms, especially convolutional neural networks (CNNs), have gradually replaced traditional machine learning methods in automatic feature extraction and high-dimensional data processing [16]. These algorithms have achieved remarkable results in tasks such as image classification, target detection, and image segmentation with their powerful learning capabilities and excellent performance, demonstrating superior results than traditional methods. Compared with traditional methods, deep learning can automatically learn more representative features from a large amount of data, overcoming the limitations of manually designing feature extractors, and showing higher robustness in complex scenarios [17]. Among numerous object detection frameworks, the YOLO series models have been widely applied in industrial inspection, autonomous driving, video surveillance, and other fields due to their excellent detection speed and high accuracy, gradually becoming the mainstream detection framework. Therefore, more and more studies have introduced them into sewer pipe defect detection tasks. Jiawei Zhang *et al*. proposed an automatic detection method for sewer pipe defects based on an improved YOLOv4, which combines spatial pyramid pooling module and DIoU loss function to improve the accuracy and efficiency of defect detection [18]. Tong Wang *et al*. proposed a detection method based on improved YOLOv5. By introducing the overlap module and GSConv simplified model into the backbone network and feature fusion network, combined with the CBAM attention mechanism, the detection accuracy of overlapping targets in complex backgrounds was improved, and knowledge distillation was used to further improve the accuracy of the model [19]. Xingliang Zhao *et al*. proposed YOLOv5-Sewer, which uses MobileNetV3 to replace the backbone network for lightweight implementation, introduces the C3-Faster module and CBAM and CA attention mechanisms to enhance feature extraction capabilities, and uses the EIOU loss function to improve defect localization accuracy [20].

Although the above methods have made some progress, there are still several limitations. First, sewer pipe images often exhibit complex backgrounds and diverse defect types. Common defects such as cracks and deposits typically have blurred boundaries and are easily confused with the background, leading to the loss of critical semantic information during feature extraction.Second, although some models have improved detection accuracy, their complex network architectures and excessive parameter counts make them unsuitable for deployment on resource-constrained embedded devices.Third, existing approaches suffer from inadequate multi-scale feature fusion and limited cross-layer semantic expression, which restricts the model’s ability to accurately detect objects at different scales and with ambiguous boundaries.To address these challenges, this paper proposes a lightweight cross-scale feature fusion model based on YOLOv8, aiming to enhance detection accuracy while significantly reducing computational cost. This design improves the model’s applicability in real-world engineering scenarios. The main contributions of this work are summarized as follows:

1. Proposal of the C2f-FAM module: Based on the traditional Bottleneck structure, the multi-scale convolution module EMSConvP is incorporated to enhance the model’s capability in extracting features across different scales, thereby improving its representation of complex texture structures and defects with blurred boundaries.
2. Design of the HS-BiFPN neck structure: A cross-layer semantic fusion path is constructed by leveraging feature interaction and re-weighting mechanisms, enabling effective integration of multi-scale features and enhancing the model’s ability to distinguish complex backgrounds and diverse defect types.
3. Introduction of the DySample dynamic upsampling mechanism: Traditional upsampling methods are replaced to improve the model’s adaptability to feature maps at varying resolutions, thereby enhancing detail preservation and optimizing overall detection performance.

## Methods

### YOLOv8

YOLOv8 [21] is a one-stage (one-stage) detector architecture, introduced by the Ultralytics team. As a major update to the YOLO (You Only Look Once) series, YOLOv8 has been fully upgraded in terms of model architecture, inference efficiency, and task scaling to significantly improve detection accuracy and speed. At the same time, YOLOv8 offers a wide range of model sizes from Nano to X-Large to meet lightweight deployment and high-performance needs. The architecture of YOLOv8 is divided into three main parts: backbone, neck, and head. The backbone is responsible for extracting features from images, the neck is responsible for feature fusion, and the head is responsible for final target prediction. The overall architecture of YOLOv8 is shown in S1 Fig.

The backbone of YOLOv8 consists of Conv, C2f, and SPPF modules, where the C2f module enhances feature representation through residual connectivity and feature fusion mechanisms while optimizing information flow and gradient transfer. Compared with the C3 module of YOLOv5 [22], C2f is more lightweight and computationally efficient. This design enables the backbone network to achieve stronger feature extraction capability with less computational overhead, providing a rich feature representation for subsequent networks.

The neck of YOLOv8 combines the FPN [23] and PAN [24] structures: the FPN combines shallow and deep features through top-down feature flow, which can retain more detailed information, while the PAN enhances semantic information transfer through bottom-up paths to optimize feature representation. This combination significantly enables the model to better capture feature information, providing strong feature support for the detection task.

The Head part is responsible for predicting the bounding box, category, and confidence of the target.YOLOv8 adopts a decoupled head structure to separate the classification and bounding box regression tasks into independent branches, which allows each task to focus on its own specific goal, thus reducing the interference between tasks, and helping to improve the performance and optimization efficiency of the model.

### Improved model architecture

Although YOLOv8 has achieved structural optimization and performance enhancement, it still faces certain challenges in pipeline defect detection scenarios. Firstly, the C2f module in its backbone network uses fixed-scale convolution kernels, lacking the ability to perceive defects of different sizes, making it difficult to extract features of defects with significant size differences in pipelines. Secondly, the feature fusion mechanism of FPN and PAN fails to fully leverage the semantic complementary relationship between shallow and deep features, resulting in limited discrimination performance when handling complex scenarios such as multiple overlapping defects or blurred boundaries. Moreover, the nearest-neighbor interpolation upsampling method used by YOLOv8 lacks the ability to dynamically adjust semantic content, which can lead to detail loss during feature map restoration and even amplify background noise, thereby reducing detection performance for targets.

In this paper, an improved YOLOv8 model is proposed, and the structure is shown in S2 Fig. The C2f-FAM module proposed in this paper introduces the EMSConvP multi-scale convolution to realize the efficient extraction of features at different scales. In addition, HS-BiFPN is designed to replace the original neck, which realizes the flexible fusion of feature maps at different scales through cross-level feature fusion operations, so that the feature maps at each scale can more fully utilize the contextual information of other scales, thus generating fine-grained feature representations, and thus enhancing the model’s multi-scale target detection capability. Finally, DySample up-sampling is introduced, which can dynamically optimize the scale of the feature map according to the size of the target and the complexity of the scene. This adaptive adjustment mechanism enables the model to capture details more effectively when facing targets of multiple sizes and different backgrounds, to improve the ability of small target detection and complex background processing.

### C2f-FAM module

In the sewer pipeline detection task, since the dataset usually contains a large number of small target objects with low resolution, the traditional convolutional layer, although enlarging the receptive field by downsampling operation, is prone to lead to the loss of the key feature information, which will have an impact on the detection accuracy. Inspired by EMCAD [25], this paper proposes an improved module, C2f-FAM, which enhances the extraction of features at different scales by introducing the EMSConvP multi-scale convolution. In this module, C2f-FAM replaces the Conv at the second position in the conventional Bottleneck structure with EMSConvP.

The EMSConvP module consists of two parts: multi-scale convolution and channel fusion layer. Firstly, multi-scale convolution kernel is used to extract the features of different receptive fields to enhance the expression ability of multi-scale targets. Subsequently, 1×1 convolution is used to adjust the number of channels and effectively fuse multi-scale features.

Multi-scale convolution uses different sizes of convolution kernels (1×1, 3×3, 5×5 and 7×7) to group the input feature maps by channel. The number of channels in each group is dynamically calculated based on the number of input channels and the number of convolution kernels, and the effectiveness of the convolution operation is ensured by limiting the number of channels in each group to no less than 16. Therefore, in this paper, we choose to replace only the third and fourth C2f modules in the backbone network. In the feature extraction process, the feature maps of each grouping independently perform the convolution operation, and the structure is shown in S3 Fig.

After the multi-scale convolution is completed, the feature maps are merged by splicing operation, the number of channels is adjusted by 1×1 convolution and the features are fused. This design realizes the fusion of multi-scale features while preserving the global semantic information, which enhances the sensitivity of the model to features of different scales. Compared with the traditional full-channel convolution, the module reduces the computational complexity and the number of parameters through grouped convolution, which reduces the computational overhead and memory occupation, and improves the computational efficiency while maintaining the performance.

The C2f-FAM module enhances the multi-scale feature extraction capability in pipeline defect detection by introducing EMSConvP, which particularly improves the detection performance for small targets. The module effectively reduces the computational complexity and the number of parameters through grouped convolution, thus optimizing the computational efficiency and memory occupation while maintaining high detection accuracy, and significantly improving the detection capability of the model.

### HS-BiFPN

To cope with the challenges of variable size, noise interference, and environmental changes in pipeline defect detection, we draw inspiration from the existing multi-scale feature fusion method HS-FPN [26] and propose an improved feature fusion neck, HS-BiFPN, which compares with the original neck of YOLOv8. BiFPN effectively improves the detection accuracy while reducing the computational and parametric quantities, and the structure is shown in S4 Fig.

HS-BiFPN is mainly composed of two parts: feature selection module and cross-level feature fusion module. The specific process is as follows: first, the different scale feature maps generated by the backbone network will be effectively filtered by the feature selection module. Subsequently, these different scale feature maps will be fused by the cross-level feature fusion module to generate features with richer semantic information. This fusion helps to capture the subtle features in the pipeline image more accurately, which in turn improves the detection capability of the model.

Feature Selection module: in the CA module, global average pooling and global maximum pooling operations are first performed on the input feature map to extract global features by calculating the average and maximum values of each channel, respectively. Global average pooling helps to obtain information evenly from the feature map, while global maximum pooling focuses on extracting the most representative data from each channel, thus minimizing information loss. With this pooling approach, the CA module can effectively capture important features from each channel and reduce the interference of redundant information. Next, the Sigmoid activation function is utilized to generate the weight values for each channel, by which each channel is weighted to enhance the representation of key features. Subsequently, a weighted feature map is generated by multiplying the computed weights with the original feature map on a channel-by-channel basis. This process allows the model to focus on more discriminative features and suppress redundant or irrelevant features, thus improving the expressiveness of the feature map. In addition, to ensure smooth cross-level feature fusion and achieve effective fusion between feature maps of different scales, a 1×1 convolution operation is used to dimensionally match feature maps of different scales, and the number of channels of each feature map is adjusted to a uniform 256, so that the feature maps can be fused in the same dimension space in the subsequent feature fusion stage, thus avoiding information loss or inconsistency due to the mismatch of channels.

Cross-Level Fusion module: in deep neural networks, the multi-scale feature maps generated by the backbone network usually contain semantic information at different levels. The high-level features usually originate from the deeper layers of the network and have high semantic richness, capturing global contextual information and abstract target features. However, due to the low spatial resolution of high-level feature maps, their ability to accurately localize targets is relatively weak, especially in the detection of tiny targets, which exhibits certain limitations. In contrast, low-level features, which usually originate from the shallow layer of the network, possess high spatial resolution and can pinpoint the location of targets and capture detailed information. However, low-level features are more limited in semantic expression, making it difficult to effectively distinguish different targets in complex scenes. Therefore, relying on high-level or low-level features alone has its shortcomings, and the fusion of the two can effectively make up for their shortcomings. By combining the advantages of high-level features and low-level features, the expression ability of the feature map can be enhanced, thus improving the accuracy and robustness of target detection.

For this reason, we propose the Cross-Level Feature Fusion (CLF) module as shown in S5 Fig. In the top-down path, high-level features are first utilized as weights for feature fusion of low-level features to enhance the semantic information of low-level features. Specifically, given a high-level feature map and a low-level feature map as inputs, the CLF module first upsamples the high-level features using a 3 × 3 convolutional kernel and a transposed convolution with a step size of two. Subsequently, the CA module processes the high-level features to generate weight values for each channel and uses these weights to filter redundant information from the low-level features. After this filtering process, the low-level features are fused with the high-level features to generate new feature maps N3, N4, and N5. Next, these fused feature maps enter the bottom-up path to perform cross-level feature fusion again. In this process, the low-level features are downsampled by a 3×3 convolution kernel and a convolution with a step size of 2. The high-level features are filtered by generating corresponding weights through the CA module. The filtered high-level features are re-fused with the low-level features. Through this series of top-down and bottom-up cross-level fusion operations, it not only effectively reduces the information loss, but also enhances the expressive ability of the feature map, and finally generates a feature map with higher semantic information and spatial resolution. In this way, the model can better capture the details of multi-scale targets, thus improving the detection accuracy in complex scenes.

### Upsample

Upsampling is a widely used technique in image processing and deep learning, aiming to convert a low-resolution image or feature map into a high-resolution output. By enlarging the image size, upsampling can effectively increase the resolution of an image or feature map, thus enhancing the model’s ability to capture detailed information. The core goal is to recover finer feature representations and provide richer feature support for subsequent processing and analysis. In YOLOv8, the traditional up-sampling method uses nearest-neighbor interpolation. This method is widely used due to its fast computational speed and simple implementation, but its limitation is that it only expands by copying the value of the nearest pixel and cannot generate new pixel information. As a result, nearest neighbor interpolation is weak in detail preservation and feature representation, especially in complex pipeline defect detection tasks, where noise interference and environmental complexity make it difficult to effectively capture subtle features and fail to meet practical needs. To overcome these problems, this paper adopts DySample [27] as the upsampling operator.

As shown in the S6 Fig, DySample first uses a point sampling generator to predict the offset of the sampling position for each pixel, then combines this with the original grid to determine the final sampling position. Then, use these positions to resample the original feature map to generate a high-resolution feature map. This process can be expressed by Eq 1. Additionally, DySample offers two methods for controlling the sampling range: one uses a fixed offset range, while the other dynamically adjusts the offset range based on the image content.

$$X^{\prime }=\text{grid\_sample}(𝒳,𝒮)$$ (1)

where 𝒳 denotes the input feature map, and 𝒮 denotes the sample set generated by the sampling point generator. The grid_sample operation resamples the input feature map 𝒳 using the sample set to generate a new feature map ${𝒳}^{\prime }$.

The sampling set 𝒮 generation process can be expressed by Eq 2:

𝒮=𝒢+𝒪 (2)

where 𝒢 denotes the original sampling grid, and 𝒪 denotes the offset.

The two point sampling generators are shown in the S7 Fig. In the static scope factor, the input features are passed through a linear layer to generate offsets 𝒪, which are then limited in range by a fixed coefficient (0.25). The offsets are then added to the original sampling grid 𝒢 after pixel blending to generate the sampling set 𝒮. In this process, 𝒪 can be expressed by Eq 3 as:

𝒪=0.25·linear(𝒳) (3)

In the dynamic range factor generator, feature maps are processed through Linear Layer 1 and the Sigmoid function to generate dynamic range factors, whose values range from 0 to 0.5. Linear layer 2 is used to generate the initial offset. This offset is then multiplied by the range factor to produce the modulated offset 𝒪. After pixel mixing, the modulated offset 𝒪 is added to the original grid 𝒢 to form the final sampling set 𝒮. In this process, 𝒪 can be expressed by Eq 4 as:

$$𝒪=0.5\cdot \sigma ({\text{linear}}_{1}(x))\cdot {\text{linear}}_{2}(x)$$ (4)

The key innovation of DySample lies in its “dynamic adjustment of sampling positions.” This can be understood as overlaying a sampling grid on an image and flexibly moving the points on the grid according to the image content to extract information from more suitable positions. This approach enables stronger adaptive capabilities during upsampling, allowing the sampling positions to be flexibly adjusted based on the local features and contextual information of the input image, thereby enhancing the detail retention capability of the upsampling results.

## Experimentation and analysis

### Datasets

The pipeline image data used in this study are sourced from the Sewer-ML [28] dataset provided by Aalborg University in Denmark, which contains approximately 1.3 million images. Because the original dataset contains a large number of duplicate or highly similar images, especially those captured consecutively under similar shooting conditions with minimal differences, we prioritized selecting samples that cover different shooting conditions, background types, lighting environments, and defect patterns to reduce redundancy and improve sample diversity. Ultimately, we selected 1,158 representative images containing six common defect categories (CK, PL, SG, SL, TL, and ZW) for this study.

To enhance the model’s generalization ability and robustness, and to ensure a roughly balanced number of samples across the six common defect categories to avoid bias toward high-frequency classes, we applied various data augmentation techniques, including geometric transformations, color adjustments, and Gaussian noise. These methods expanded the dataset to 1,952 images, which were then split into training, validation, and test sets at a ratio of 8:1:1. The number and proportion of images for each defect category before and after augmentation are shown in Table 1. Additionally, during model training, we employed online data augmentation using Mosaic augmentation, which effectively increases image diversity and further improves the model’s robustness and detection accuracy. Examples of pipeline defect images are shown in S8 Fig.

**Table 1:** Defect type and distribution before and after augmentation.

Type	Description	Label Number	Before Augmentation	After Augmentation
CK	Misalignment	0	257 (22.20%)	355 (18.19%)
PL	Crack	1	195 (16.84%)	342 (17.51%)
SG	Tree Roots	2	196 (16.92%)	333 (17.06%)
SL	Leakage	3	131 (11.31%)	302 (15.47%)
TL	Detachment	4	172 (14.85%)	288 (14.76%)
ZW	Obstacle	5	207 (17.88%)	332 (17.01%)

### Experimental environment settings

The experiments in this paper were conducted on a system running Ubuntu 22.04. The hardware configuration consists of a 12th generation Intel(R) Core(TM) i7-12700F processor with a base clock of 2.10 GHz and an NVIDIA GeForce RTX 4090 graphics card. The software environment uses Python 3.10, and the deep learning framework is PyTorch 2.4.1. detailed configuration information of the experimental platform is shown in Table 2.

**Table 2:** Configuration information of the experimental platform.

Configuration	Versions
CPU	Intel(R) Core(TM) i7-12700F
GPU	NVIDIA GeForce RTX 4090
RAM	64G
Python	3.10
CUDA	12.2
PyTorch	2.4.1

The experiments were conducted for 300 training periods using an input image size of 640 × 640 pixels. The optimizer was set to SGD, the momentum was set to 0.937, the initial learning rate was set to 0.01, and the weight decay was set to 0.0005. The detailed experimental parameters for model training are shown in Table 3.

**Table 3:** Experimental parameters of the training model.

Parameter	Value
Image size	640*640
Batch size	32
Epochs	300
Optimizer	SGD
Momentum	0.937
Initial learning rate	0.01
Weight Decay	0.0005

### Evaluation indicators

In order to comprehensively evaluate the performance of our model, we adopt precision (P), recall (R), mean accuracy (mAP), number of parameters (Params, M), floating-point operations (FLOPs, G), detection speed (Speed, ms), and model size (Size, MB) as the evaluation criteria.

Precision (P) Measures the proportion of samples predicted to be in the positive category that are truly positive by the model. It is defined as the ratio between true positives (TP) and all samples predicted to be in the positive category (i.e., the sum of true positives and false positives (FP)). The formula for calculating the precision is shown in Eq 5.

$$P=\frac{TP}{TP+FP}$$ (5)

where TP is the number of true positive predictions and FP is the number of false positive predictions. High precision indicates that the model is less likely to incorrectly predict negative class samples as positive.

Recall, also known as sensitivity or true positive rate, is used to assess the ability of the model to recognize all relevant positive samples. It is defined as the ratio between true positives (TP) and all actual positive samples (i.e., the sum of true positives and false negatives (FN)). The formula for recall is shown in Eq 6.

$$R=\frac{TP}{TP+FN}$$ (6)

where TP is the number of true positive predictions and FN is the number of false negative predictions. A high recall indicates that the model is able to identify most of the positive samples that are actually present.

Mean Average Precision (mAP) is a widely used metric in object detection to summarize the precision-recall tradeoff. It is calculated by averaging the average precision (AP) across all classes. Average Precision for each class is computed as the area under the precision-recall curve. The formula for mAP is shown in Eq 7.

$$mAP=\frac{1}{N}\sum_{i=1}^{N}AP_{i}$$ (7)

where N is the number of classes, and APi is the average precision for class i.

In addition, in order to evaluate the efficiency of the model, this paper chooses the number of parameters, floating-point operations, detection speed and model size as evaluation criteria. The number of parameters reflects the total number of weights and biases to be learned by the model during the training process, which is an important measure of model complexity and is related to memory and storage requirements. The amount of computation, expressed in FLOPs, measures the number of floating-point operations required for a single inference, and is crucial for evaluating the efficiency of the model, especially on embedded or mobile devices. Detection speed measures the performance of the model in real-time applications and directly affects the real-time requirements in industrial inspection, autonomous driving and edge computing. Model file size reflects the storage requirements of the model, and optimizing the file size is critical for memory footprint and transfer efficiency on resource-constrained devices such as embedded systems and mobile devices.

### Ablation experiment

To visualize the impact of the various improvements proposed in this paper on the model performance, ablation experiments are conducted in this subsection. The experiment uses YOLOv8n as the baseline model introduces the C2f-FAM, HS-BiFPN, and DySample modules sequentially for improvement, and presents the corresponding experimental results. Specific results can be found in Tables 4 and 5.

**Table 4:** The results of ablation experiment.

Baseline	C2f-FAM	HS-BiFPN	DySample	Precision(%)	Recall(%)	mAP(%)
YOLOv8n	×	×	×	88.8	68.6	79.8
	✓	×	×	81.5	77.7	82.2 ↑2.4
	×	✓	×	80.4	76.6	81.4 ↑1.6
	×	×	✓	91.6	71.9	82.1 ↑2.3
	✓	✓	×	88.1	71.4	82.5 ↑2.7
	✓	✓	✓	90.5	75.2	83.6 ↑3.8

**Table 5:** The results of model efficiency metrics in ablation experiment.

Baseline	C2f-FAM	HS-BiFPN	DySample	Params(M)	FLOPs(G)	Speed(ms)	Size(MB)
YOLOv8n	×	×	×	3.01	8.1	3.07	5.98
	✓	×	×	2.94	8.0	3.15	5.87
	×	✓	×	2.08	7.1	3.15	4.24
	×	×	✓	3.02	8.1	3.00	6.01
	✓	✓	×	2.02	7.0	3.26	4.13
	✓	✓	✓	1.95	6.4	3.13	4.01

To investigate the effect of each improved module on model accuracy, we added each module to the baseline model one by one, and the experimental results are shown in Table 4. First, we analyzed the effect of each individual module on model performance. Compared with the baseline model, the introduction of the C2f-FAM module recall increased by 9.1% and mAP increased by 2.4%. The C2f-FAM module enhances target feature capture through multi-scale feature extraction, which, in turn, improves recall and precision. Replacing the original neck structure with HS-BiFPN recall increased by 8% and mAP increased by 1.6%. HS-BiFPN improves the model’s ability to adapt to targets of different scales through enhanced multi-scale feature fusion, thereby improving detection performance for targets of various sizes. Upsampling with DySample precision increased by 2.8%, recall increased by 3.3%, and mAP increased by 2.3%. The DySample module enhances detail retention by dynamically adjusting offsets during upsampling. When these three modules were introduced simultaneously, precision increased by 1.7%, recall increased by 6.6%, and mAP increased by 3.8%. The experimental results demonstrate that the proposed improved model offers better detection performance in pipeline defect detection tasks.

Table 5 shows that C2f-FAM and HS-BiFPN effectively reduce the number of parameters and FLOPs to different degrees, especially HS-BiFPN, where the number of parameters decreased from 3.01M to 2.08M, and FLOPs decreased from 8.1G to 7.1G. Meanwhile, DySample maintains nearly the same number of parameters and FLOPs while improving the accuracy. Finally, our improved model the number of parameters reduced from 3.01M to 1.95M, the FLOPs reduced from 8.1G to 6.4G, and the model size reduced from 5.98MB to 4.13MB achieving significant reductions in terms of the number of parameters, FLOPs, and model size.

To enhance the statistical reliability of the experimental results, we conducted five independent training runs for each model configuration using different random seeds. The resulting mAP values were then compared with those of the baseline model using paired t-tests to evaluate whether the performance improvements introduced by each module were statistically significant. The statistical results are presented in Table 6.

**Table 6:** Statistical test results.

Baseline	C2f-FAM	HS-BiFPN	DySample	Shapiro-Wilk p	t-test p
YOLOv8n	×	×	×	–	–
	✓	×	×	0.425	0.0004
	×	✓	×	0.272	0.0001
	×	×	✓	0.858	0.0001
	✓	✓	×	0.251	0.0002
	✓	✓	✓	0.437	0.0001

Prior to the t-tests, we applied the Shapiro–Wilk test to assess the normality of the mAP differences for each comparison. The results indicated that all comparison groups satisfied the normality assumption (Shapiro-Wilk p > 0.05), with Shapiro–Wilk p-values of 0.425 for C2f-FAM, 0.272 for HS-BiFPN, 0.858 for DySample, 0.251 for C2f-FAM + HS-BiFPN, and 0.437 for the full model. These results validate the applicability of the paired t-test.

The subsequent paired t-tests demonstrated that all module configurations significantly outperformed the baseline model in terms of mAP, with statistically significant differences (t-test p < 0.05). Specifically, the t-test p-values were 0.0004 for C2f-FAM, 0.0001 for HS-BiFPN, 0.0001 for DySample, 0.0002 for C2f-FAM + HS-BiFPN, and 0.0001 for the full model.

These results provide strong statistical evidence that the performance improvements brought by the proposed modules are significant and not attributable to random fluctuations.

### Five-fold cross-validation

To evaluate the generalization ability of the model in the task of underwater pipeline defect detection, this study adopts a five-fold cross-validation approach. As a commonly used statistical method, cross-validation provides a reliable estimate of model performance through repeated training and validation, while effectively reducing evaluation bias caused by the randomness of data partitioning.

As shown in S9 Fig, the original dataset is randomly divided into five mutually exclusive subsets of equal size. In each validation cycle, one subset is used as the validation set, while the remaining four subsets form the training set. The network is trained iteratively on the training set and evaluated on the validation set. This five-fold cross-validation method enables a comprehensive evaluation across different data partitions, effectively reducing the risk of overfitting and enhancing the reliability of model performance assessment.

The results of the five-fold cross-validation experiment are shown in Table 7. The model exhibits relatively stable performance under different partitions, with average Precision, Recall, and mAP reaching 89.7%, 75.1%, and 83.1%, respectively, which are relatively close to the original fixed partition test results (90.5%, 75.2%, and 83.6%), indicating that the model does not overly rely on specific data partitions.

**Table 7:** Five-fold validation results.

K-Fold	Precision(%)	Recall(%)	mAP(%)
Fold 1	90.3	75.8	83.9
Fold 2	91.8	74.0	84.1
Fold 3	87.2	78.1	84.4
Fold 4	90.8	74.9	82.8
Fold 5	88.4	72.5	80.5
Average	89.7	75.1	83.1

### Comparison experiment

Table 8 shows the results of comparing the performance of the baseline model YOLOv8 with the improved model. As shown in the table, our model outperforms the baseline model in several key metrics, with precision increased by 1.7% (from 88.8% to 90.5%), recall increased by 6.6% (from 68.6% to 75.2%), and mAP50 increased by 3.8% (from 79.8% to 83.6%). In addition, the number of parameters in the improved model decreased by 1.06M (from 3.01M to 1.95M), FLOPs decreased by 1.7G (from 8.1G to 6.4G), and the model size decreased by 1.97MB (from 5.98MB to 4.01MB). Although the inference speed slightly increased by 0.06ms (from 3.07ms to 3.13ms), this minor cost is outweighed by the significant performance improvements.

**Table 8:** Performance comparison between YOLOv8 and our model.

Model	Precision(%)	Recall(%)	mAP(%)	Params (M)	FLOPs (G)	Speed (ms)	Size (MB)
YOLOv8	88.8	68.6	79.8	3.01	8.1	3.07	5.98
Ours	90.5	75.2	83.6	1.95	6.4	3.13	4.01

To further evaluate the detection performance of our proposed model, we perform a comprehensive comparison with other target detection algorithms. The algorithms involved in this comparison include YOLOv5 [22], YOLOv10 [29], YOLOv11 [21], Faster R-CNN [30], RT-DETR [31], Swin Transformer [32], DINO [33], TOOD [34] and Deformable-DETR [35].

In our experiments, all baseline models were trained from scratch on the same training dataset as the proposed model, using a consistent data split strategy for both training and evaluation. To ensure the fairness of the comparative experiments, we applied identical training configurations to all models, including input image size, optimizer type, learning rate, momentum, and weight decay (Table 2 for details). In addition, all experiments were conducted under the same hardware and software environment (configuration details are provided in Table 3) to eliminate performance deviations caused by platform differences. These measures collectively ensure the reproducibility and credibility of the comparative results.

Table 9 shows the detailed performance comparison of the 10 models on the dataset. Our model achieves leading performance in five of the seven metrics, including Precision, Recall, mAP, number of parameters, and model size. Specifically, our model achieves higher Precision (90.5%), Recall (75.2%), and mAP (83.6%) on the dataset. Meanwhile, the number of model parameters is only 1.95M and the model size is only 4.01MB.

**Table 9:** Performance comparison of different models on the dataset.

Model	Precision(%)	Recall(%)	mAP(%)	Params (M)	FLOPs (G)	Speed (ms)	Size (MB)
YOLOv5	87.8	75.1	80.7	7.03	15.8	3.31	13.7
YOLOv10	89.0	65.3	77.1	2.27	6.5	3.07	5.5
YOLOv11	78.5	73.6	80.6	2.58	6.3	3.03	5.23
Faster R-CNN	72.0	60.6	67.0	41.37	184.3	14.66	319
RT-DETR	87.2	69.1	77.1	28.46	100.6	5.49	56.3
Swin-Transformer	83.8	74.9	82.3	69.11	318.5	29.67	797
DINO	79.8	56.9	66.9	47.55	243.7	27.40	575
TOOD	74.2	55.4	66.0	32.03	174.1	17.70	245
Deformable-DETR	67.1	67.0	69.5	40.1	171.0	21.10	472
Ours	90.5	75.2	83.6	1.95	6.4	3.13	4.01

Compared with the other models in the table, our model shows a remarkable balance between efficiency and accuracy. Compared with Swin Transformer, which is ranked second in mAP, our model has 67.16M fewer parameters, 312.1G fewer FLOPs, and 792.99MB fewer model size, while the detection accuracy improves by 1.3% and the inference speedup is 26.54ms. These results show that our model achieves a better balance between detection accuracy and computational efficiency, and can realize high-precision target detection while maintaining real-time detection capability.

### Inference performance evaluation on NVIDIA Jetson AGX Orin

To evaluate the model’s actual deployment performance on an embedded platform, this study selected the NVIDIA Jetson AGX Orin (64GB) as the test device. We used TensorRT to accelerate the model deployment, converting the trained PyTorch format ".pt" file into a TensorRT-supported ".engine" file.

We evaluated the model performance under both FP32 and FP16 modes, with the results shown in Table 10. The detection accuracy of the model remained nearly identical across the two precision settings. Under FP32, the average inference time was 18.9 milliseconds with a model size of 10.2 MB. In contrast, the FP16 mode reduced the inference time to 16.5 milliseconds and decreased the model size to 7.7 MB.An inference speed of 16.5 milliseconds per frame corresponds to approximately 60.6 frames per second (FPS), indicating the model’s capability for real-time object detection on embedded platforms.

**Table 10:** Inference results of our model with different precision types.

Model	Precision Type	Precision(%)	Recall(%)	mAP(%)	Speed (ms)	Size (MB)
Ours	FP32	91.7	71.9	83.1	18.9	10.2
	FP16	91.6	71.9	83.0	16.5	7.7

### Visualization of experimental results

In order to visually demonstrate the effectiveness of the improved model compared with the baseline model, this paper carried out a visual analysis of the experimental results, as shown in S10 Fig. In S10 Fig(a–c), it can be observed that the baseline model has inaccurate target localization in some scenarios, while the improved model can locate the defects more precisely. In S10 Fig(e–f), the confidence level of the improved model for defects is significantly improved (from 0.53 to 0.88). In addition, in S10 Fig(g–i), the baseline model fails to detect the CK defect labeled 0, while the improved model successfully identifies the defect; in S10 Fig(j–l), the baseline model misidentifies the SL defect labeled 3 as the SG defect labeled 2, while the improved model can correctly classify the defect.

To further visually verify the effectiveness of the proposed improved model, this paper generates a heat map of the detection results of the baseline model and the improved model through the Grad-CAM visualization technique. In the heat map, different colors indicate the strength of the model’s attention. The red area indicates that the model pays more attention to the location, which is the area that the model considers most likely to contain defects; while the green and blue areas indicate that the model pays less attention. This color distribution can intuitively reflect the model’s attention focus and responsiveness to the defective region, and the results are shown in S11 Fig

From S11 Fig, it can be seen that the baseline model’s attention region is relatively dispersed and has limited ability to focus on defects. In S11 Fig(a–c), the distribution of attention in the heat map of the baseline model is relatively dispersed and fails to accurately focus on the core location of defects. In contrast, the thermograms of the improved model show more concentrated red areas and can focus more accurately on the defect core location. In S11 Fig(g–i), it can be observed that the thermogram of the baseline model exhibits broken and discrete characteristics with discontinuous attention distribution, while the improved model has a more complete and continuous attention distribution. In addition in S11 Fig(d–f,j–l), the improved model’s attention thermogram has a more comprehensive coverage of the defective region.

In summary, the proposed improved model exhibits a more accurate attentional focusing ability in the defect detection task, which can effectively capture the key features of defects and reduce the interference of background noise. In addition, the improved model provides a more complete and continuous response to the defective region in complex environments, which significantly improves the accuracy and robustness of detection. This experimental result fully verifies the effectiveness of the improved model in the pipeline defect detection task.

## Conclusion

Aiming at the problems faced by defect detection in sewer pipeline, this paper proposes a lightweight cross-scale feature fusion sewer pipeline defect detection model. We design the C2f-FAM module and HS-BiFPN neck, and introduce the DySample upsampling operation, so that the model can efficiently extract features at different scales and better capture the details of multi-scale targets, thereby enhancing its ability to resolve fine details in defect regions and improving the accuracy of semantic discrimination. Experimental results show that the improved model has significant advantages in detection accuracy and lightweighting. Specifically, our model achieves 90.5%, 75.2%, and 83.6% in precision, recall, and mAP metrics, respectively, demonstrating better detection results. In addition, the model only has 1.95M number of parameters, 6.4G FLOPs, and 4.01M model size, which effectively reduces the number of parameters and FLOPs of the model while improving the precision, and possesses the potential to be applied in pipeline defect detection.

In addition, we conducted deployment experiments on the NVIDIA Jetson platform. The results show that the model maintains excellent performance on embedded platforms: accuracy of 91.6%, recall of 71.9%, mAP of 83.0%, average detection speed of 16.5 ms (approximately 60.6 FPS), and model size of 7.7 MB. This demonstrates that the model not only achieves excellent detection performance but also possesses the capability for real-time inference on resource-constrained devices.

Although this study achieved good performance on the current dataset, the detection model may face the following main challenges in actual engineering applications: First, the sewer pipe environment is often accompanied by significant uncertainty, such as internal corrosion and sediment obstruction in pipes, morphological differences caused by diverse pipe structures, changes in lighting, medium obstruction, and changes in perspective caused by the movement of detection equipment. These complex factors may interfere with the model’s detection accuracy; Second, existing datasets may not cover all types of pipeline defects and their complex background environments. Therefore, the model may perform below expectations when faced with more diverse pipeline defects or more complex environments. Additionally, during actual deployment, engineering issues such as the model’s operational efficiency, resource consumption, and real-time performance on embedded devices must be considered, all of which impose higher requirements on the model’s practical applicability.

To further enhance the model’s adaptability and engineering application value, future research will focus on the following areas. First, multi-source data collection strategies will be introduced, combined with advanced sample generation methods such as diffusion models, to enhance the diversity of training data in terms of categories and environmental conditions, thereby improving the model’s detection capabilities in complex scenarios and for rare defects. Second, transfer learning and domain adaptation techniques will be explored to promote the model’s generalization ability across different geographical regions, pipeline types, and data collection conditions, enabling it to adapt to more variable and complex real-world environments while reducing reliance on labeled data. Finally, efforts will continue to optimize the model structure and deployment process, with a focus on lightweight design and inference speed improvement. Techniques such as model quantization and pruning will be integrated to enhance real-time performance and stability on resource-constrained embedded platforms.

## Supporting information

S1 Fig Structure of YOLOv8. (TIF)

S2 Fig Improved model architecture. (TIF)

S3 Fig EMSConvP. (TIF)

S4 Fig HS-BiFPN. (TIF)

S5 Fig CLF. (TIF)

S6 Fig Architecture of DySample. (TIF)

S7 Fig Sampling point generator. (TIF)

S8 Fig Example of dataset. (TIF)

S9 Fig Five-fold cross-validation. (TIF)

S10 Fig Results of detection. (TIF)

S11 Fig Heatmap results of detection. (TIF)
