# Supplementary figures and images for: A lightweight cross-scale feature fusion model based on YOLOv8 for defect detection in sewer pipeline
Source: PLoS One. 2025 Aug 21;20(8):e0330677. doi: 10.1371/journal.pone.0330677 (PMC12370142; doi:10.1371/journal.pone.0330677)

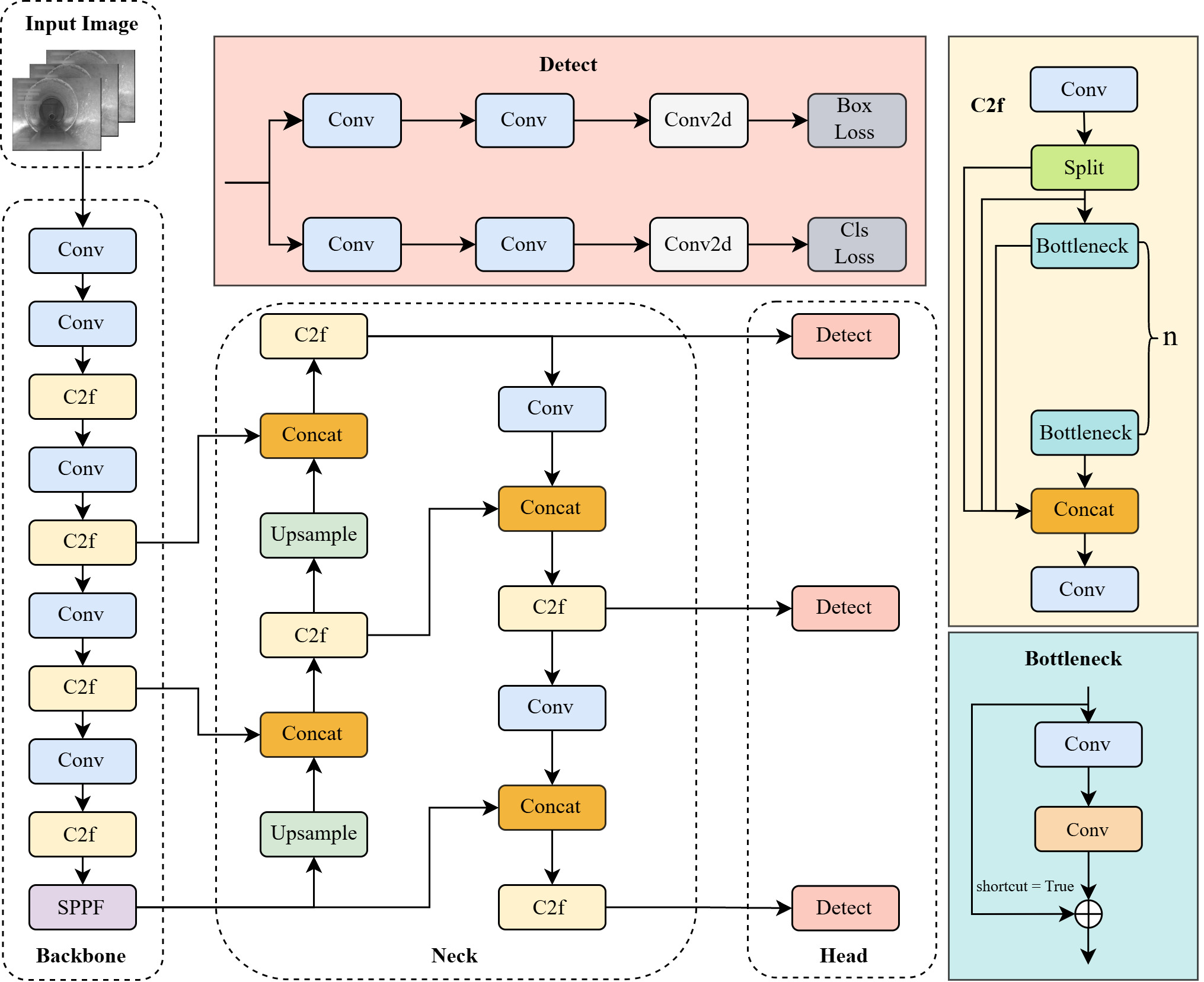

Supplement: S1 Fig — (TIF) [file pone.0330677.s001.tif]

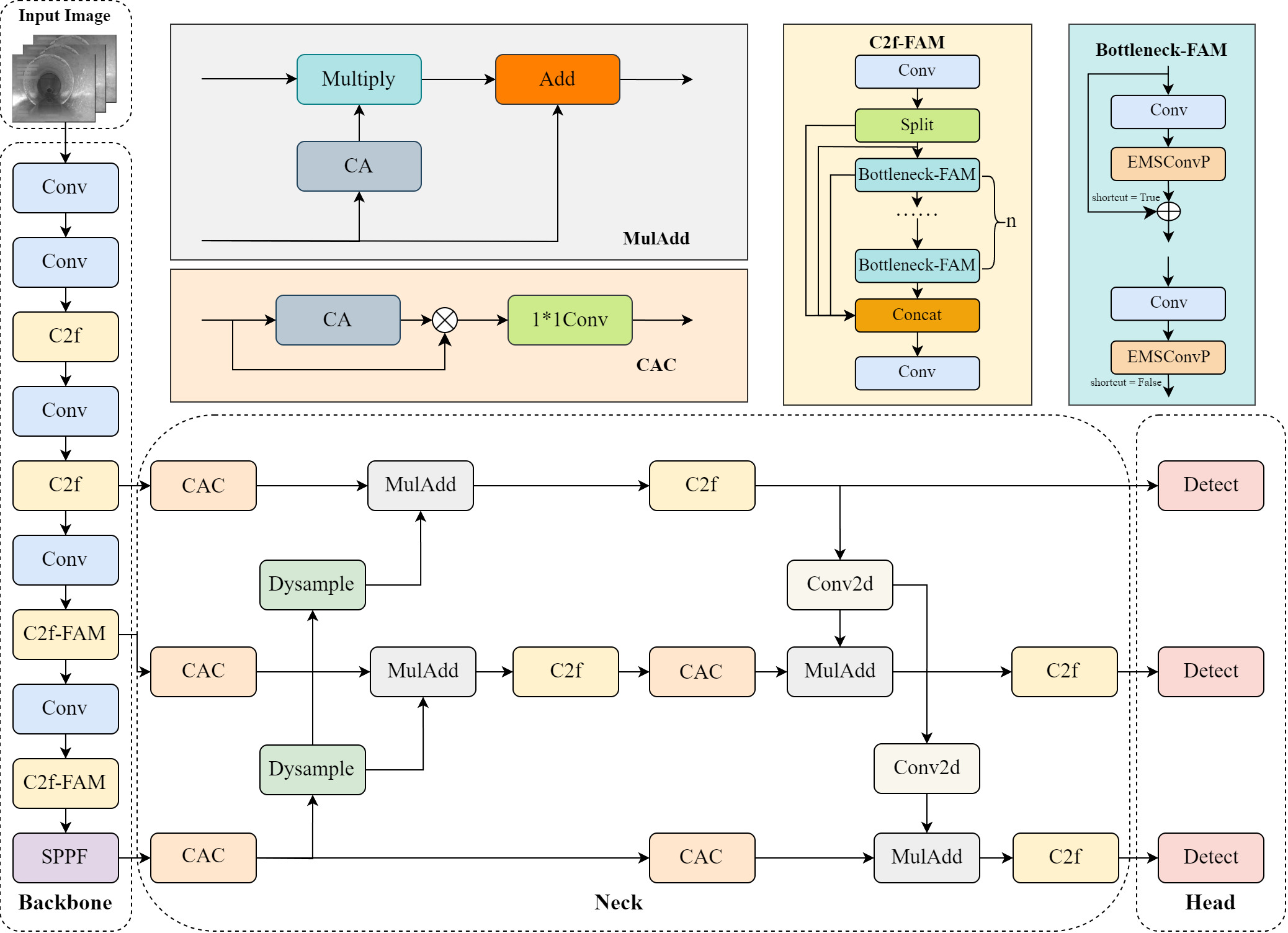

Supplement: S2 Fig — (TIF) [file pone.0330677.s002.tif]

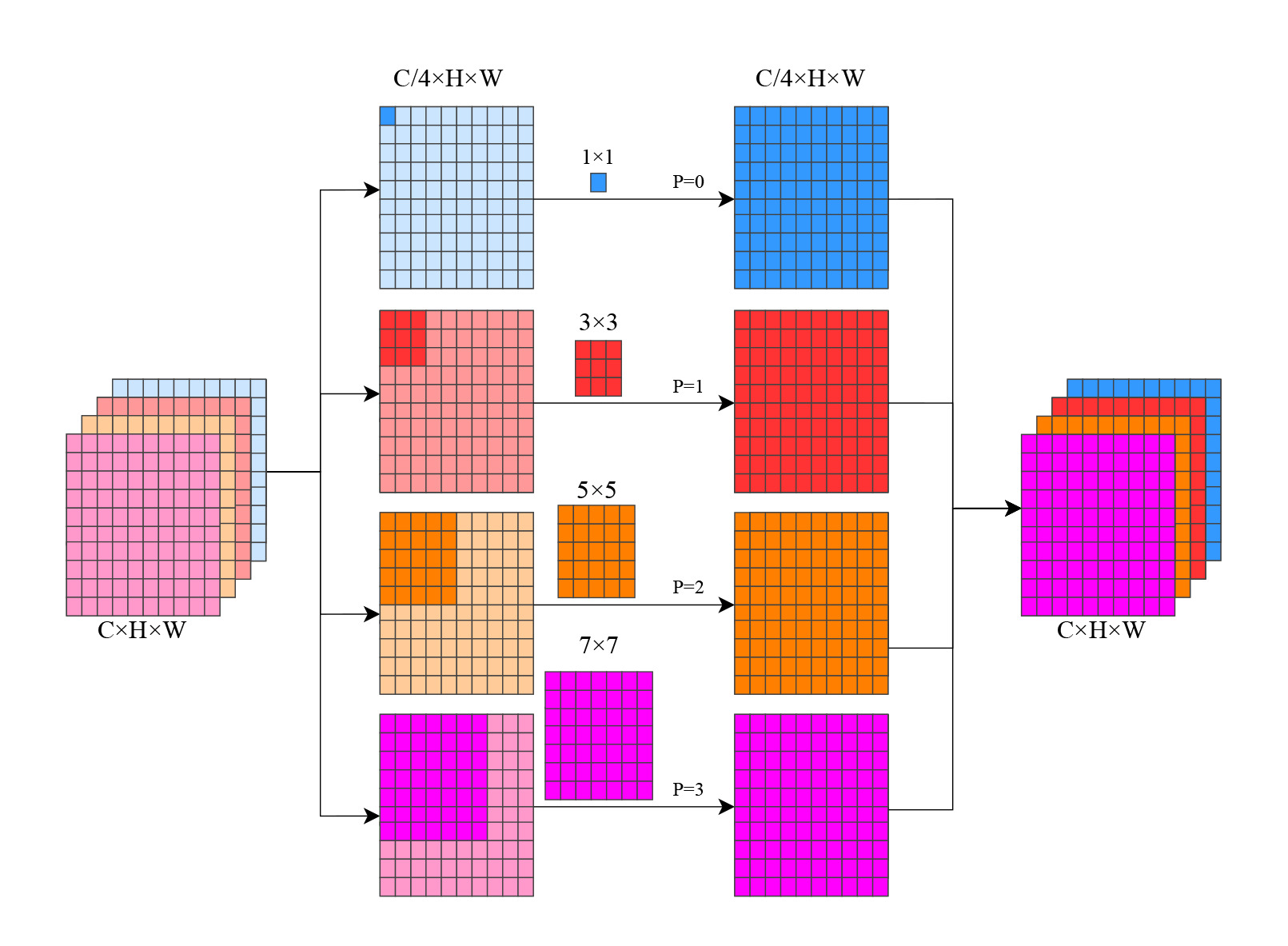

Supplement: S3 Fig — (TIF) [file pone.0330677.s003.tif]

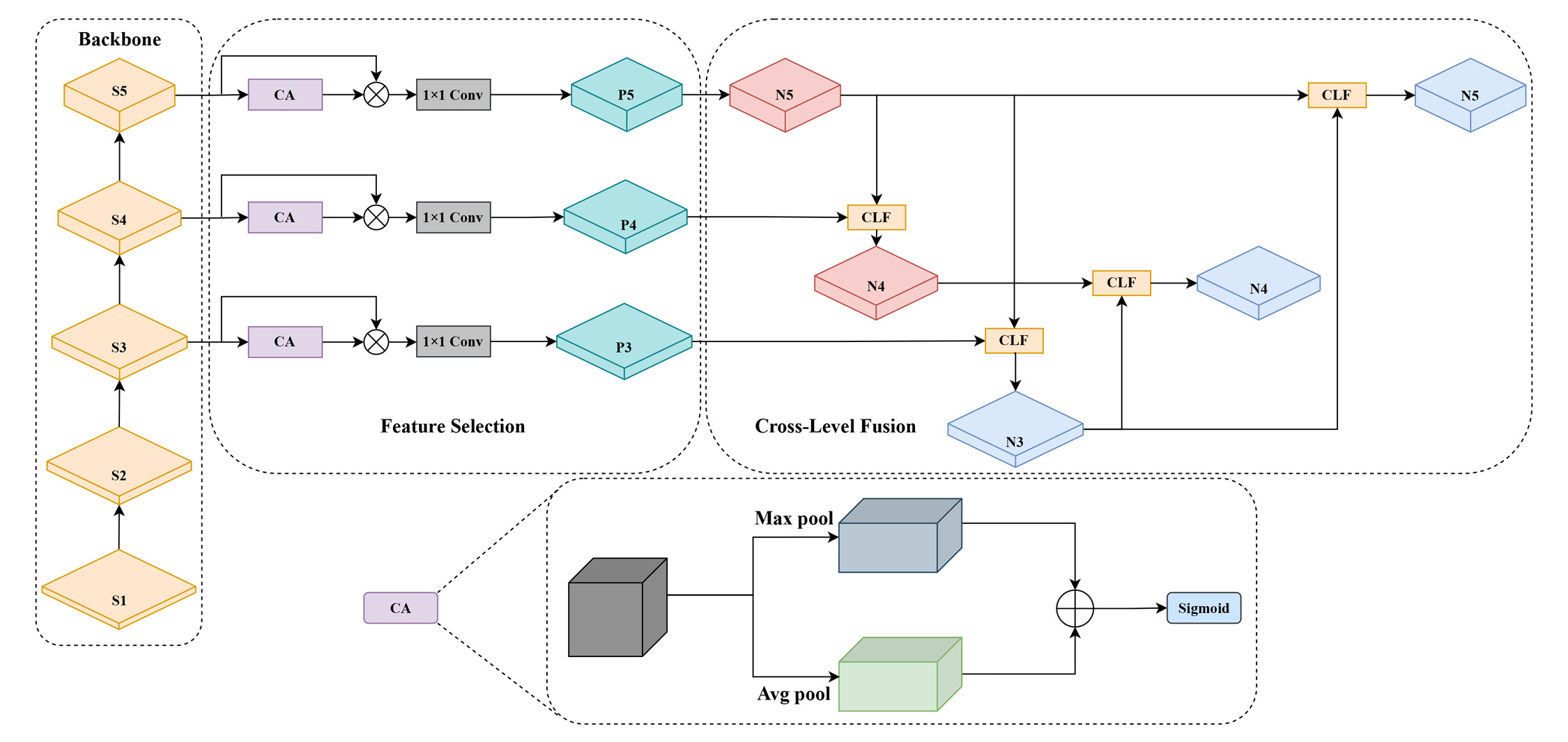

Supplement: S4 Fig — (TIF) [file pone.0330677.s004.tif]

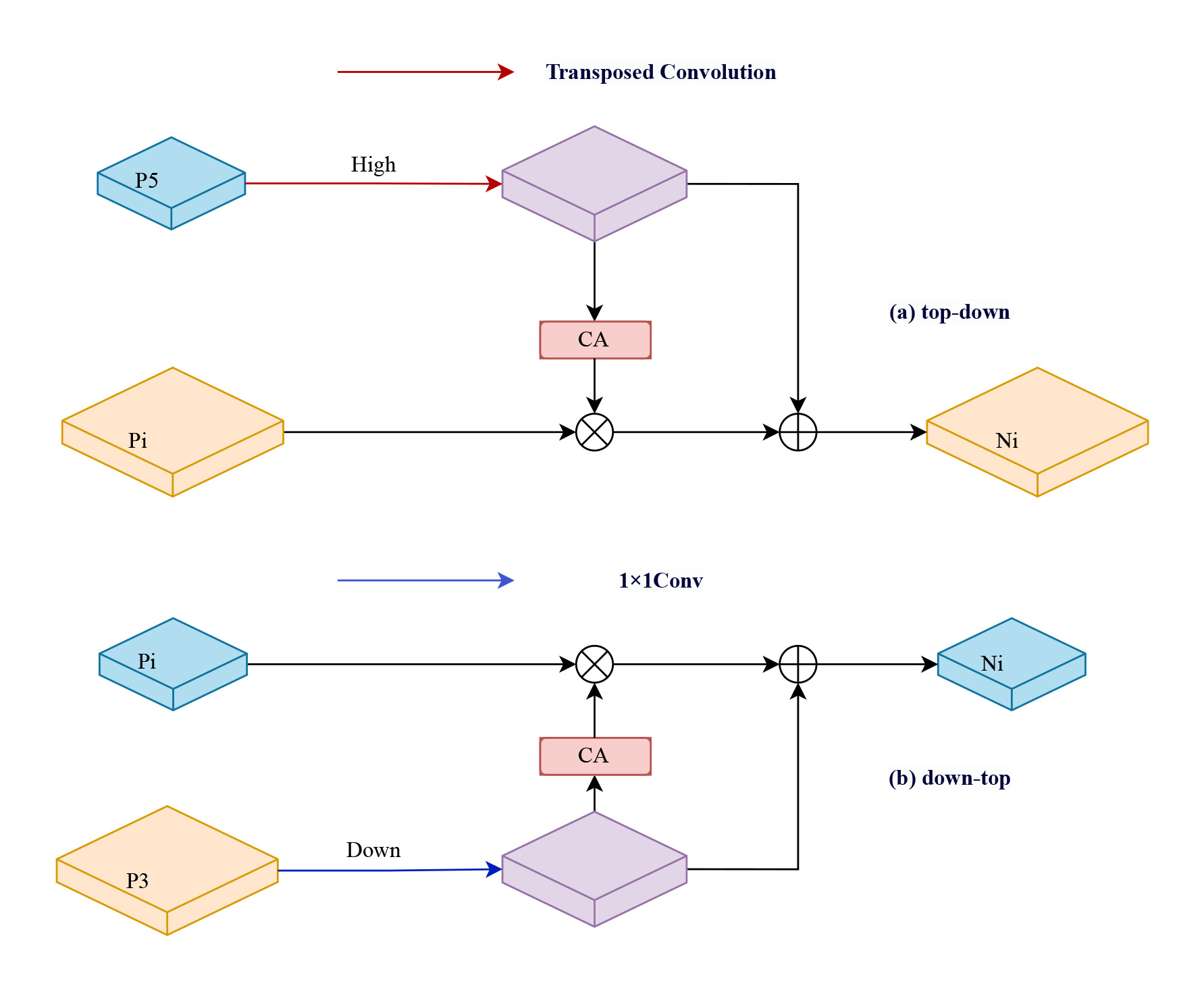

Supplement: S5 Fig — (TIF) [file pone.0330677.s005.tif]

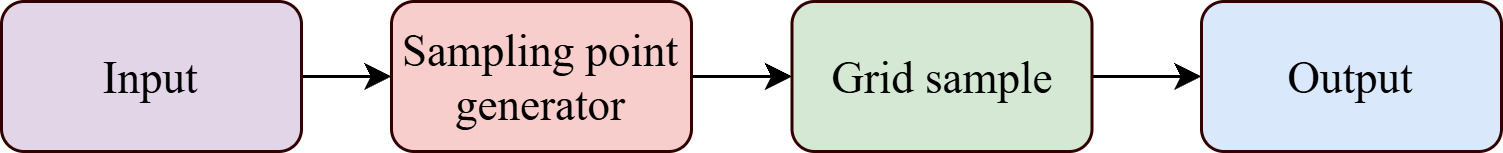

Supplement: S6 Fig — (TIF) [file pone.0330677.s006.tif]

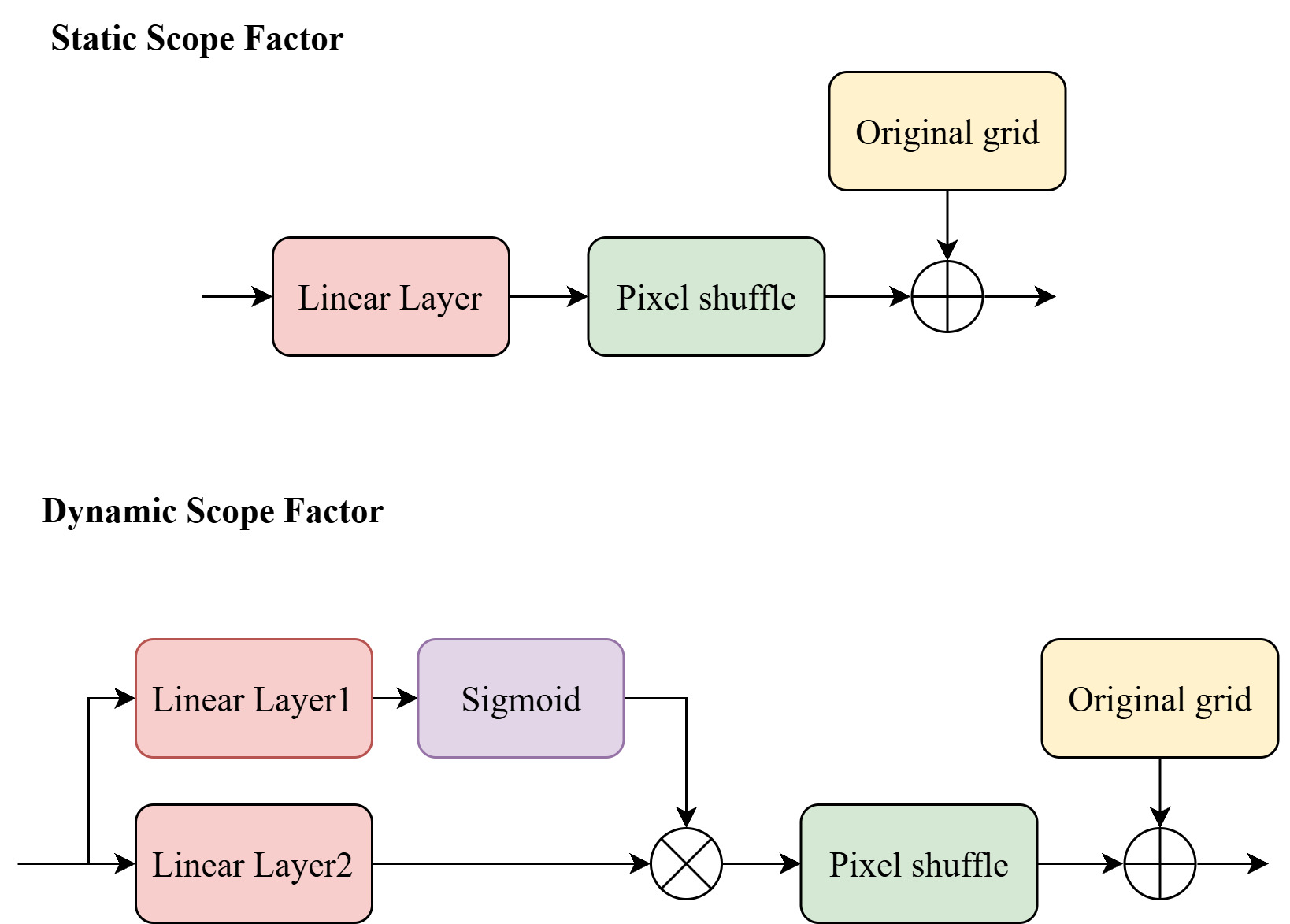

Supplement: S7 Fig — (TIF) [file pone.0330677.s007.tif]

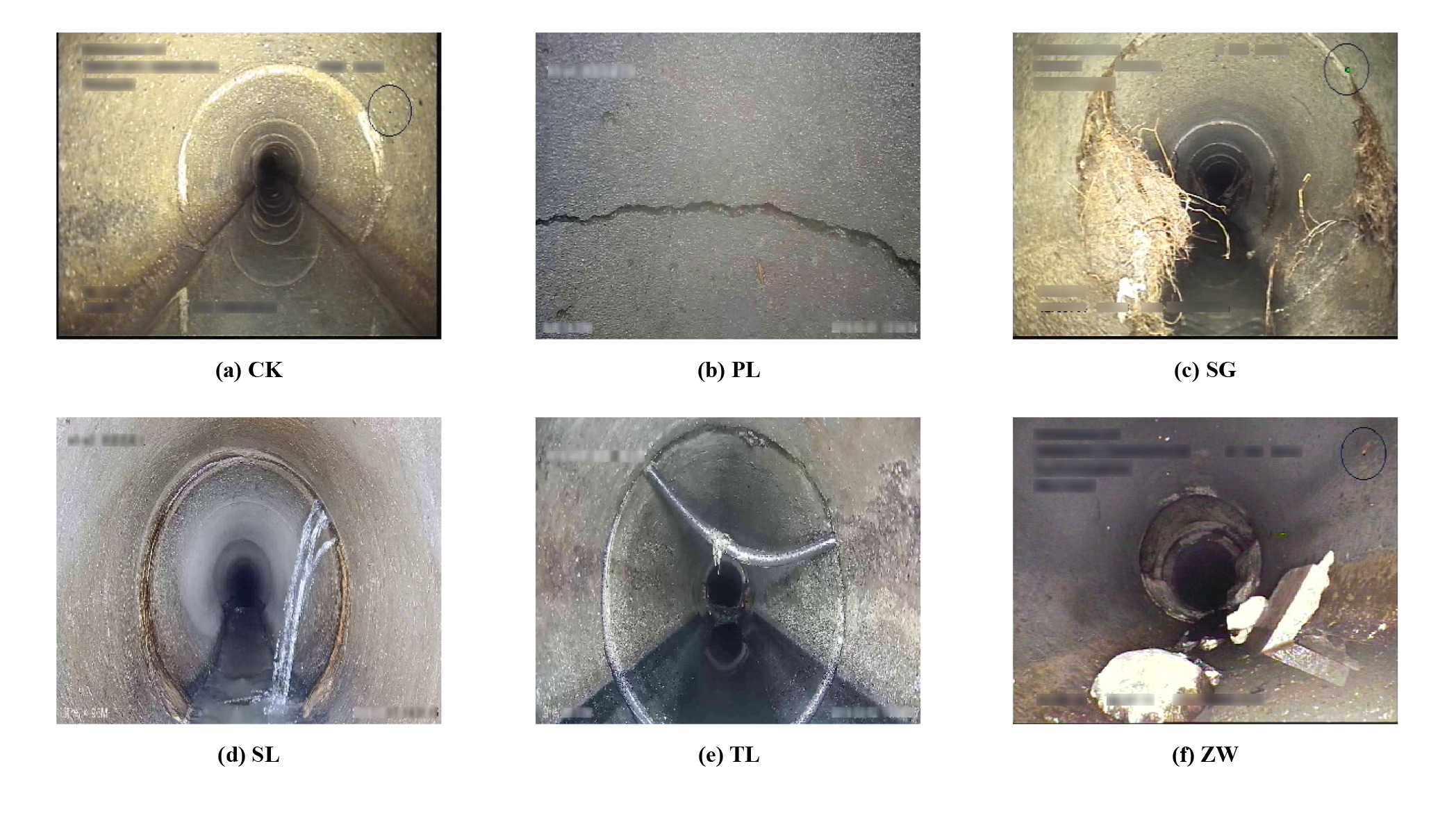

Supplement: S8 Fig — (TIF) [file pone.0330677.s008.tif]

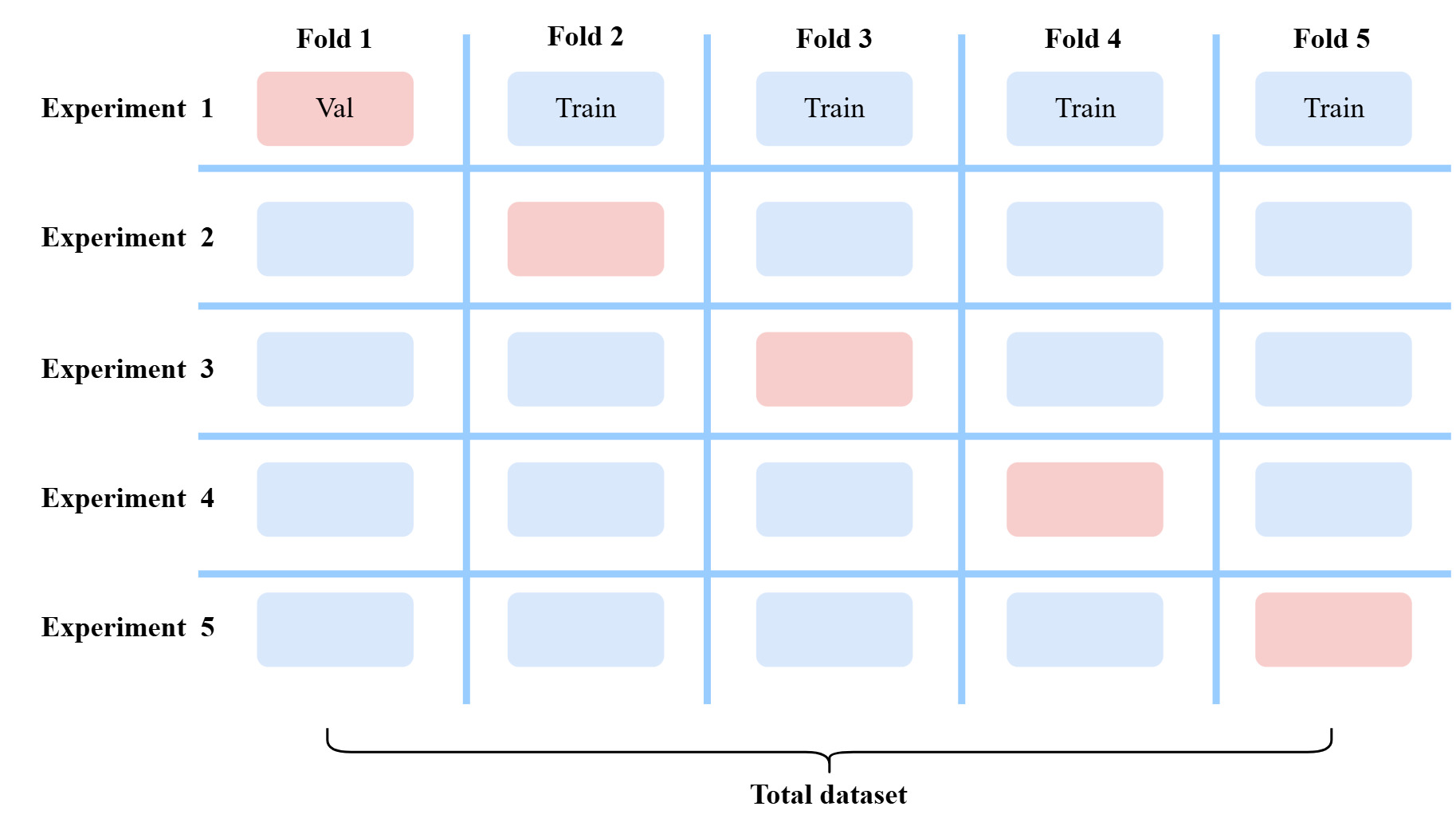

Supplement: S9 Fig — (TIF) [file pone.0330677.s009.tif]

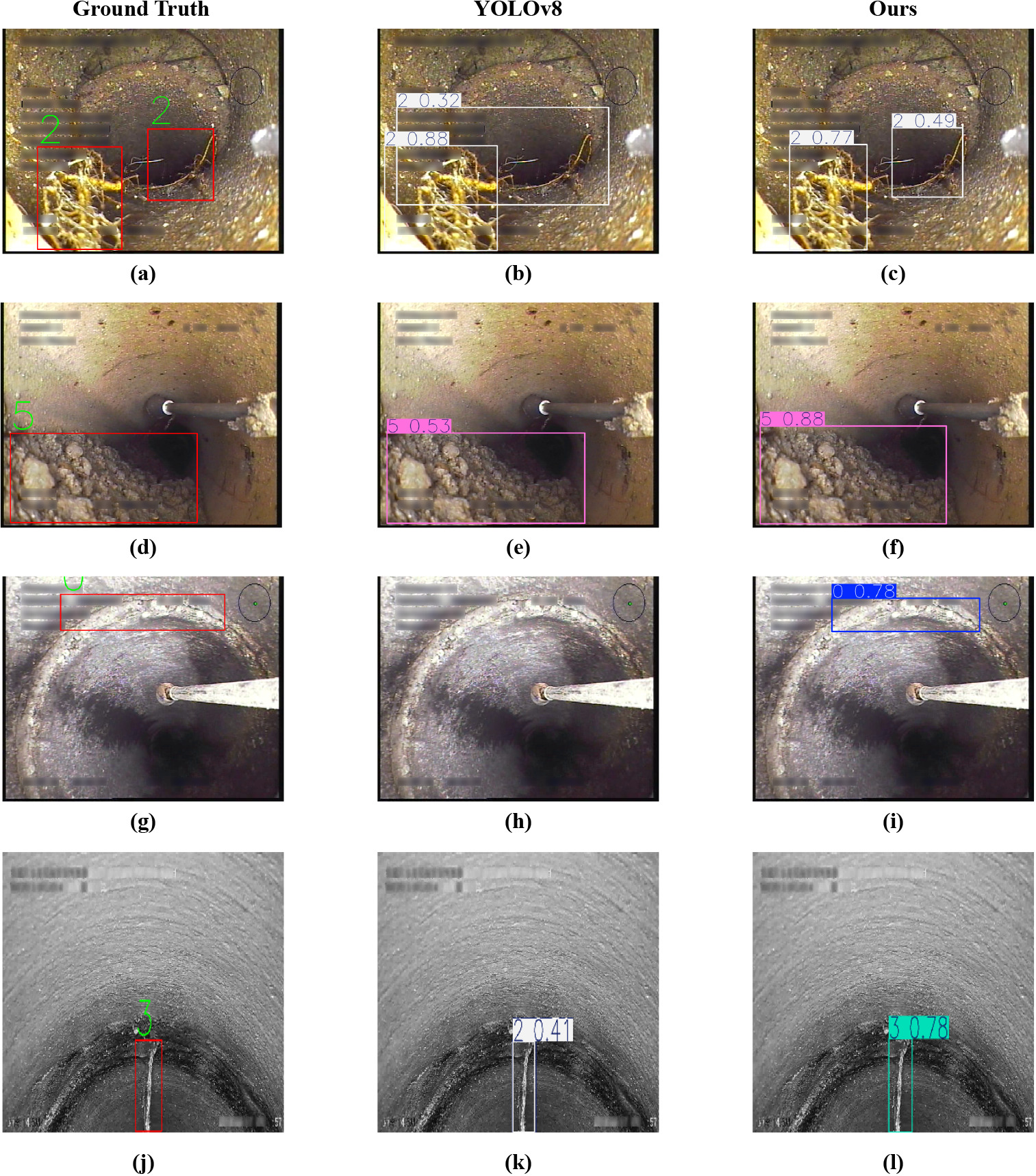

Supplement: S10 Fig — (TIF) [file pone.0330677.s010.tif]

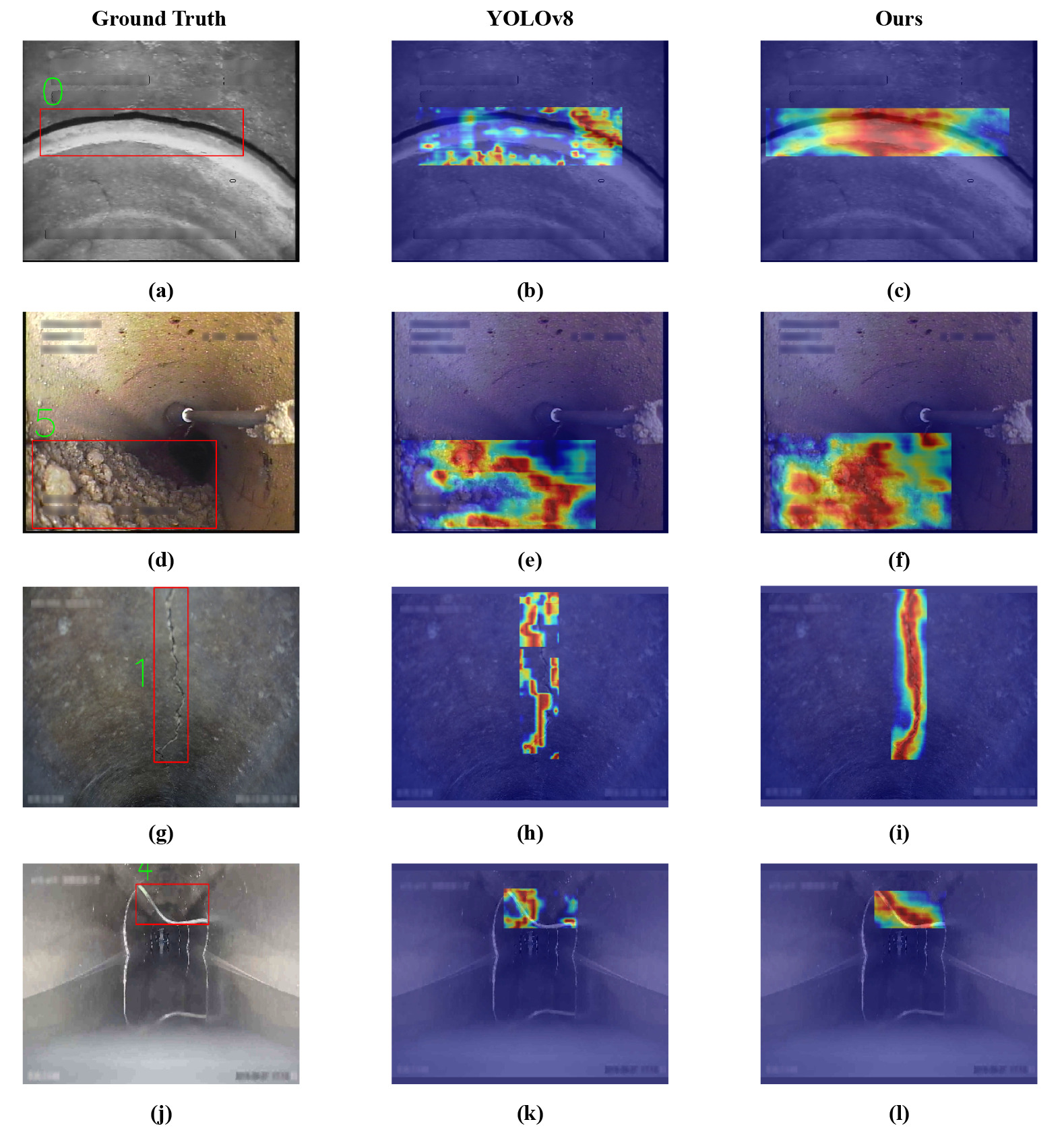

Supplement: S11 Fig — (TIF) [file pone.0330677.s011.tif]
